# Equity in oncology care: addressing disparities in cancer treatment in Georgia

**DOI:** 10.3389/fpubh.2024.1381075

**Published:** 2024-05-02

**Authors:** Amany R. Keruakous, Inemesit Akpan, Michael Chahin, Aghapy Kirolos, Mai Keruakous

**Affiliations:** ^1^Hematology Oncology Department, Georgia Cancer Center, Wellstar MCG Health, Augusta, GA, United States; ^2^Piedmont Athens Regional, Athens, GA, United States; ^3^Baylor College of Medicine, Houston, TX, United States; ^4^Mercer County Community College, Trenton, NJ, United States

**Keywords:** racial and ethnic differences, cancer care access, equity, policy recommendation, community intervention

## Abstract

This research delves into the disparities in access to oncology care among cancer patients in Georgia, with a specific focus on the distinct challenges faced by African American (AA) individuals compared to non-African American (Non-AA) counterparts. Leveraging data from the 2020 Behavioral Risk Factor Surveillance System (BRFSS) survey and supplementary online resources, the study meticulously examines socioeconomic factors, including income, education, and insurance coverage, which significantly influence the quality of cancer care received. The analysis reveals substantial income gaps between AA and Non-AA patients, underscoring the critical implications for healthcare access. Moreover, AA patients exhibit lower rates of full insurance coverage for cancer-related treatments, posing additional barriers to comprehensive care. By investigating the intersections of race, income, and education, the research aims to pinpoint the root causes of these disparities and proposes evidence-based solutions to address the identified challenges. The ultimate objective is to contribute valuable insights that inform targeted policy recommendations and community-based interventions, fostering a more equitable landscape for oncology care in Georgia. This study seeks to amplify awareness and advocate for tangible measures, striving toward healthcare equity for all cancer patients, irrespective of their racial or socioeconomic backgrounds.

## Introduction

In the state of Georgia, it is anticipated that 61,170 new cancer cases will have been diagnosed in 2023, with an estimated 18,510 mortality (1). The African American (AA) population in Georgia represents roughly one-third of the state’s total population, a significant contrast to the national average of 14% (2). Despite these demographic differences, cancer survival rates among African Americans in Georgia tend to be notably lower than those among their non-African American counterparts for nearly all types of cancer (3). It is essential to recognize that these disparities in health outcomes cannot be viewed in isolation.

There are multiple potential factors affecting cancer-related outcomes. Wagner, et al. identified worse mortality-to-incidence ratios for black patients in comparison to white patients for multiple cancer types. The contrast was most evident in prostate, cervical, and men with oral cancer. A striking finding of this study is the impact of geographic location within the state on these mortality figures. For example, both racial groups, had higher mortality-to-incidence rations in southwest and south central regions of Georgia compared to the Atlanta area (4).

The issue appears to be even more complex, however. For example, Luningham, et al. reviewed over 19,000 cases of patients with breast cancer and found higher rates of mortality in black women compared to white women. They found an association with residence in a more deprived area, based on an area deprivation index, with poorer outcomes for both cohorts. Curiously, living in an area with a lower deprivation score was associated with better outcomes for white women, but not necessarily for black women (5).

Another population-based study reviewed cancer specific mortality for breast, colorectal, lung, and prostate cancers at the county level for the state of Georgia. Notably, counties that were shown to be “hot spots” for cancer mortality were not necessarily the same across the four cancer types. County characteristics included race and ethnicity, age, sex, obesity, tobacco use, education, household income, and access to healthcare. Certain counties were found to have higher cancer-related mortality and this was associated, overall, with a higher non-Hispanic black population, age, poverty level, and rural status (6).

This inequality in cancer care outcomes raises critical questions regarding the influence of racial disparities on the delivery of healthcare services in Georgia. It prompts a deeper examination of the systemic and structural factors that contribute to the imbalance in cancer survival rates and the overall health of the African American population in the state. Based on available studies, considerations such as availability of healthcare access, environmental exposures, tobacco use, public awareness of cancer screenings, and health insurance coverage come to mind. Understanding the root causes and mechanisms behind these challenges is crucial for developing effective strategies to ensure equitable access to healthcare and improve health outcomes for all residents of Georgia, regardless of their racial or ethnic background (Table 1).

**Table 1:** Summary of socioeconomic characteristics for all oncology respondents to the 2020 BRFSS survey: State of Georgia.

Factor	All respondents in GA = 9,090 Oncology patients: 400 (4.4%)	
	Count	Percent
**Gender**		
Male	148	37
Female	252	63
**Health care coverage**		
Yes	384	96
No	14	3.5
**Not see physician due to cost**		
Yes	32	8
No	368	92
**Education level**		
Less than high school	35	8.8
High school graduate	216	54.3
College graduate	147	36.9
**Employment status**		
Employed	72	18.1
Un-Employed	325	81.9
**Annual income category**		
Less than 25k	82	26.1
25–50k	91	29
More than 50 k	141	55
**Race/Ethnicity**		
Non-African American	335	84.2
African American	63	15.8
**Health insurance pay for cancer treatment**		
Yes	153	96.8
No	5	3.2

## Methods

We harnessed the data from the 2020 Behavioral Risk Factor Surveillance System (BRFSS) (7) to gather patient-reported information encompassing a range of demographic characteristics and health coverage status.

The Behavioral Risk Factor Surveillance System (BRFSS) questionnaire was developed in collaboration between CDC and public health departments in the following states, the District of Columbia, Puerto Rico, and the US Virgin Islands. Data derived from the questionnaire provides health departments, public health officials, and policymakers with behavioral information that, when combined with mortality and morbidity statistics, inform public health officials as they establish health-related policies and priorities and address and assess strategies to promote good health. The BRFSS is designed to provide state-level estimates and measure progress in health care outcomes (Table 2).

**Table 2:** Comparison of socioeconomic characteristics between oncology respondents of African American descent and non-African American descent to the 2020 BRFSS survey: State of Georgia.

Factor	AA	Non-AA	*p*-value
	%	%	
**Gender**			
Male	36.5	37.3	0.9034
Female	63.5	62.7	
**Health care coverage**			
Yes	95.2	96.1	0.7012
No	4.8	3.2	
**Not see physician due to cost**			
Yes	11.1	7.4	0.1147
No	88.8	92.6	
**Education level**			
Less than high school	11.3	8.1	0.4384
High school graduate	58.1	53.6	
College graduate	30.7	38.3	
**Received summary of cancer treatment**			
Yes	66.7	64.8	0.1830
No	33.3	35.2	
**Received written instructions**			
Yes	89.5	83	0.2338
No	10.5	17	
**Ever denied insurance coverage because of cancer**			
Yes	11.1	6.6	0.2018
No	88.9	93.4	
**Participated in cancer clinical trial**			
Yes	0	6.1	0.2411
No	100	93.9	
**Health insurance paid for all cancer treatment**			
Yes	84	99.3	0.0022
No	16	0.7	
**Employment status**			
Employed	17.5	18.4	0.1407
Un-Employed	82.5	81.6	
**Annual income category**			
Less than 25k	40.4	23.3	0.0151
25–50 k	31.9	28.5	
More than 50k	27.7	48.1	

Additional analysis: Increased likelihood of full insurance coverage to all cancer treatment expenses among participants with higher educational level (high school and college grads) *p* = 0.0206. Non-AA oncology patients in the state of Georgia are 4 times more likely to have full coverage for cancer related treatment than AA oncology patients (OR = 4.31).

BRFSS is administered through phone calls made randomly to sample representatives of adult residents aged 18 years or older in all 50 states in the US, the District of Columbia, and the three US territories. BRFSS completes more than 400,000 adult interviews each year, making it the largest continuously conducted health survey system globally. The questionnaire consists of an annual standard core, a biannual rotating core, optional modules, and state-added questions.

BRFSS respondents are identified through telephone-based methods, such as landline and cellular telephone calls. Adding cellular telephone calls to the survey has improved the validity, response rate, and representativeness of BRFSS data. Iterative proportional fitting (or “raking”) is a new weighting methodology that has replaced the post stratification method to weight BRFSS data. Raking allows incorporation of cellular telephone survey data and permits additional demographic characteristics and age-race/ethnicity-gender to be integrated.

Specifically, our study focused on individuals diagnosed with oncological conditions in the state of Georgia. Our primary objective was to investigate how racial disparities impact the delivery of clinical services within this patient population.

### Statistical analysis

We analyzed pooled cross-sectional data from the Behavioral Risk Factor Surveillance System (BRFSS) 2020. Data were analyzed to estimate health care disparities of respondents to the 2020 BRFSS survey. We measured specific health care disparities for all respondents in the state of Georgia, including patient’s gender, race/ethnicity, health care coverage, education level, employment status, annual income, history of cancer, and delays in medical care due to financial burden, among respondents. We computed the difference between our comparison groups, AA vs. non-AA, using a chi-square test for categorical variables and a t-test for continuous variables. Demographic factors were analyzed through weighted analysis for accessibility to medical care.

Further, we performed a multivariate analysis computing the impact of different socioeconomic disparities such as employment status and education level and their correlation with health care insurance coverage.

By utilizing the wealth of data available through the BRFSS, we sought to gain a comprehensive understanding of the interplay between demographic factors, health coverage, and the care received by oncology patients in Georgia. Our analysis was dedicated to shedding light on the extent to which racial disparities affect the types and quality of clinical services that these patients access. This research is invaluable for pinpointing areas of inequality and crafting targeted interventions to promote equitable healthcare access and improve the overall well-being of oncology patients in the state.

## Results

In the state of Georgia, the 2020 Behavioral Risk Factor Surveillance System (BRFSS) survey drew responses from 9,090 individuals, with 400 of them being oncology patients (comprising 4.4% of the total sample). Among these oncology patients, 37% were male, and 63% were female. A majority of these respondents reported having health care coverage (96%), and 96.8% had insurance coverage for all cancer-related treatments, even though a notable 81.9% of participants were unemployed.

When comparing African American (AA) respondents to non-African American (Non-AA) respondents, distinct differences emerged in various aspects of their socioeconomic profiles. AA participants reported lower rates of health insurance payment for cancer treatment (84% versus 99.3%, with a *p*-value of 0.0022) and lower annual incomes (with 72.3% earning less than $50,000 per year compared to 51.5% among Non-AA participants, with a p-value of 0.0151). The odds ratio for full insurance coverage for all cancer treatment expenses was 4.31, indicating that AA participants were four times less likely to have such comprehensive coverage compared to Non-AA participants.

However, it is important to note that there were no statistically significant differences between AA and Non-AA participants in terms of secondary education rates, health care coverage, the ability to see a physician without cost barriers, receipt of a summary of cancer treatment or written instructions, denial of insurance coverage due to a cancer diagnosis, and participation in cancer clinical trials.

Additionally, a noteworthy finding from this analysis was that participants with at least a secondary education were more likely to have full insurance coverage for all cancer treatment expenses, with a *p*-value of 0.0206.

In summary, the data indicates that there are disparities in healthcare access and coverage among oncology patients in Georgia, particularly with regard to insurance coverage for cancer treatment and income levels, with African American patients facing a greater burden in this regard. Addressing these disparities is crucial to ensure equitable access to healthcare and improved health outcomes for all oncology patients in the state.

## Discussion

The data analysis of oncology patients in Georgia reveals a pressing issue: significant disparities in access to high-quality cancer care between African American (AA) and non-African American (Non-AA) patients. These disparities are rooted in variations in income levels and insurance coverage, which have far-reaching implications for the overall quality of care and health outcomes for these individuals. This discussion will explore the challenges posed by these disparities and propose solutions to overcome them.

## Challenges

### Income disparities

The data underscores a stark income gap between AA and Non-AA oncology patients in Georgia, with a substantially higher percentage of AA patients falling into the lower income brackets. Low income can lead to financial barriers in seeking timely and appropriate cancer care, such as out-of-pocket expenses and travel costs to healthcare facilities (8). This could also explain the lower quality of life they experience as survivors (9). Low income patients can face severe financial hardship due to the cost of treatment that sometimes requires them to make choices between health care and other necessities, which contributes to poor health outcomes (10).

### Insurance coverage disparities

AA patients were found to have less reported full insurance coverage for cancer-related treatment. Inadequate insurance coverage can result in patients forgoing essential treatments, delaying care, late stage diagnosis, or being burdened with significant medical bills (11). This, in turn, affects the overall quality and efficacy of cancer treatment. Patients with inadequate insurance are less likely to have preventative health screening and with limited access to primary care for prompt evaluation of symptoms, they are more likely to be diagnosed with advanced stages of cancer (10, 12). Insurance and cost related barriers remain prevalent due to decline in coverage by employee sponsored health insurance and the continuously rising costs of medical care (10). The topic of health insurance coverage is controversial and the type of coverage available can vary by state. For example, in Georgia, Medicaid expansion under the Affordable Care Act was not adopted. Instead, there is the Georgia Pathways to Coverage program. While not intended to review the merits of this program as compared to others, the minimal work hours and lack of retroactive coverage may increase the cost burden for many patients (13). This potential connection is shown by the number of respondents in the 2020 BRFFS survey who are unemployed and uninsured.

### Educational disparities

The analysis indicates that individuals with at least a secondary education were more likely to have full insurance coverage. This highlights the importance of education in understanding and navigating the complexities of the healthcare system (14, 15). Educational disparities can hinder access to essential resources and information, impacting health-seeking behaviors and decision-making. Limited health literacy may limit understanding of preventative health screening and may affect discussions pertaining to treatment and need for follow up, thereby, predisposing to late stage presentation and management of cancer. It is also associated with decreased adherence with treatment plan and decreased participation in clinical trials (16).

#### Cultural barriers

Limitation in culturally appropriate cancer and treatment related information makes it a challenge for patients with different cultural backgrounds to navigate cancer care. It results in our affected population being less informed about treatment options and how to best navigate cancer care (17). Cultural barriers further contributes to mistrust of the health system, poor communication between patients and physicians, and in perceived societal stigma of certain cancers (18).

#### Geographic disparities

There is concern for worsening cancer care disparities among rural population possibly due to limited access to newest prevention, diagnosis, and treatment services. Clinicians with very subspecialized skillsets have been found to cluster in urban communities thereby adversely affecting outcomes of patients with certain cancers in rural communities (19). Further limitation to cancer care for patients in rural communities has been associated with lack of adequate public transportation and lack of resources to pay for transportation to oncological facilities. This highlights how longer distance to larger diagnostic centers contributes to low screening rates, increased time to diagnosis, and poorer outcomes of cancer is more prevalent in patients living in rural communities (20).

## Potential solutions

### Income and employment initiatives

Implement and expand income support programs, such as Medicaid expansion, to ensure that individuals from lower-income backgrounds have access to affordable healthcare services (21). Additionally, promoting employment opportunities and job training can help uplift individuals and families economically.

### Comprehensive insurance coverage

Enhance healthcare policies to ensure comprehensive insurance coverage for all cancer treatments, regardless of racial or ethnic background. This may involve revising insurance regulations, expanding Medicaid eligibility, and reducing out-of-pocket costs for patients (10, 22).

### Educational outreach

Develop targeted educational programs that focus on health literacy and navigating the healthcare system. These programs should be accessible to individuals with diverse educational backgrounds and tailored to the specific needs of different communities. Additionally, provide resources to help patients understand their insurance options and rights.

Healthcare professionals caring for patients with limited health literacy should use easy to understand terminologies, communicate with pictures/interactive programs for education, and use either a “teach back” or “show me” method to assess understanding (16, 23).

### Community-based healthcare initiatives

Establish community health centers and clinics in underserved areas, particularly those with a high proportion of AA residents. These centers can provide essential cancer screening, prevention, and treatment services, as well as education on early detection and lifestyle factors that affect cancer risk (24, 25).

### Culturally competent care

Promote cultural competence among healthcare providers to ensure that patients receive care that is sensitive to their unique cultural and socioeconomic circumstances (26). Encourage diversity in the healthcare workforce and provide training to reduce bias in healthcare delivery. It is important to proactively partner with disadvantaged communities to get better insights on barriers to care and to help implement sustainable solutions that will improve outcomes of cancer care (27).

### Policy advocacy

Engage in advocacy efforts at the state and national levels to address the structural factors that contribute to disparities in healthcare access and outcomes. These efforts can include pushing for policies that promote economic and educational equity and comprehensive healthcare reform (28).

The disparities in oncology care identified in Georgia are multifaceted and deeply entrenched in socioeconomic and racial factors. Information collected from the BRFSS database is limited in that it helps identify areas of inequality without necessarily revealing the root cause, and is at the scope of the state level. However, these data, along with that collected by other epidemiologic studies of the population in Georgia can be used to improve access to healthcare for higher risk groups. A top to bottom approach, from the federal to local government level is needed to address these issues.

Overcoming these challenges requires a comprehensive approach that combines economic, educational, and healthcare policy interventions. By addressing the root causes of these disparities, we can move closer to ensuring that all individuals, regardless of their racial or ethnic background, have equitable access to high-quality oncology care and ultimately improve cancer outcomes for everyone in the state of Georgia.

## Conclusion

In the context of cancer patients in Georgia, African American (AA) patients were to found have overall lower income levels compared to their non-African American (Non-AA) counterparts. Furthermore, they were found to lower reporting of comprehensive insurance coverage for cancer-related treatments. An in-depth analysis of the data indicates that having at least a secondary education is associated with having full insurance coverage.

These disparities in income and educational attainment among cancer patients may have a substantial impact on their ability to access and receive high-quality cancer care. The interplay between education and income disparities, in particular, may contribute to the unequal distribution of resources and opportunities in the realm of oncology care.

To ensure that all cancer patients, regardless of their racial or ethnic background, have access to high-quality oncology care, it is crucial to address these disparities at a societal level. Implementing policies and initiatives that promote educational opportunities and income equality is vital for creating a healthcare system that is more equitable and inclusive, ultimately leading to improved health outcomes for all individuals affected by cancer in Georgia.

Differences in socioeconomic and health coverage for AA and non-AA respondents to the 2020 BRFSS survey: State of Georgia.

**Table:** 

	Factor	African American (AA) (%)	Non-AA (%)	*p*-value
Health care coverage	Yes	95.2	96.1	0.7012
	No	4.8	3.2	
Participated in cancer clinical trial	Yes	0	6.1	0.2411
	No	100	93.9	
Health insurance paid for all cancer treatment	Yes	84	99.3	0.0022
	No	16	0.7	
Annual income	Less than 25 k	40.4	23.3	0.0151
	25–50 k	31.9	28.5	
	More than 50 k	27.7	48.1	

## Data availability statement

The original contributions presented in the study are included in the article/supplementary material, further inquiries can be directed to the corresponding author.

## Author contributions

ARK: Conceptualization, Data curation, Formal analysis, Funding acquisition, Investigation, Methodology, Project administration, Resources, Software, Supervision, Validation, Visualization, Writing – original draft, Writing – review & editing. IA: Validation, Writing – original draft, Writing – review & editing. MC: Writing – review & editing. AK: Writing – review & editing. MK: Writing – original draft, Writing – review & editing.

## References

[1] American Cancer Society. Cancer statistics center. Available at: http://cancerstatisticscenter.cancer.org/

[2] United States Census Bureau. (2022). Available at: https://www.census.gov/quickfacts/fact/table/GA/.

[3] Cancer Facts and Figures. Atlanta: American Cancer Society. (2023). Available at: https://www.cancer.org/content/dam/cancer-org/research/cancer-facts-and-statistics/annual-cancer-facts-and-figures/2023/2023-cancer-facts-and-figures.pdf.

[4] Wagner SE Hurley DM Hébert JR McNamara C Bayakly AR Vena JE. Cancer mortality‐to‐incidence ratios in Georgia. Cancer. (2012) 118:4032–45. doi: 10.1002/cncr.26728, PMID: 22294294 PMC3342438

[5] Luningham JM Seth G Saini G Bhattarai S Awan S Collin LJ. Association of race and area deprivation with breast cancer survival among black and white women in the state of Georgia. JAMA Netw Open. (2022) 5:e2238183. doi: 10.1001/jamanetworkopen.2022.38183, PMID: 36306134 PMC9617173

[6] Moore JX Tingen MS Coughlin SS O'Meara C Odhiambo L Vernon M. Understanding geographic and racial/ethnic disparities in mortality from four major cancers in the state of Georgia: a spatial epidemiologic analysis, 1999-2019. Sci Rep. (2022) 12:14143. doi: 10.1038/s41598-022-18374-7, PMID: 35986041 PMC9391349

[7] Center for Disease Control and Prevention. 2020 BRFSS survey data and documentation. (2022). Available at: https://www.cdc.gov/brfss/annual_data/annual_2020.html.

[8] Moss JL Pinto CN Srinivasan S Cronin KA Croyle RT. Persistent poverty and cancer mortality rates: an analysis of county-level poverty designations. Cancer Epidemiol Biomarkers Prev. (2020) 29:1949–54. doi: 10.1158/1055-9965.EPI-20-0007, PMID: 32998949 PMC7534551

[9] Short PF Mallonee EL. Income disparities in the quality of life of Cancer survivors. Med Care. (2006) 44:16–23. doi: 10.1097/01.mlr.0000188986.84819.3a 16365608

[10] Ward E Halpern M Schrag N Cokkinides V DeSantis C Bandi P. Association of insurance with cancer care utilization and outcomes. CA Cancer J Clin. (2008) 58:9–31. doi: 10.3322/CA.2007.0011 18096863

[11] Zhao J Han X Nogueira L Fedewa SA Jemal A Halpern MT. Health insurance status and cancer stage at diagnosis and survival in the United States. CA Cancer J Clin. (2022) 72:542–60. doi: 10.3322/caac.21732 35829644

[12] Roetzheim RG Pal N Tennant C Voti L Ayanian JZ Schwabe A. Effects of health insurance and race on early detection of Cancer. J Natl Cancer Inst Monogr. (1999) 91:1409–15. doi: 10.1093/jnci/91.16.1409 10451447

[13] Gordon SH Cole MB Huberfeld N. Georgia pathways-partial medicaid expansion with work requirements and premiums. JAMA. (2023) 330:1225–6. doi: 10.1001/jama.2023.15811, PMID: 37713204

[14] Barcelo A Duffett-Leger L Pastor-Valero M Pereira J Colugnati FAB Trapido E. The role of education on Cancer amenable mortality among non-Hispanic blacks & non-Hispanic whites in the United States (1989–2018). BMC Cancer. (2021) 21:907. doi: 10.1186/s12885-021-08633-7 34493242 PMC8425171

[15] Albano JD Ward E Jemal A Anderson R Cokkinides VE Murray T. Cancer mortality in the United States by education level and race. J Natl Cancer Inst Monogr. (2007) 99:1384–94. doi: 10.1093/jnci/djm127 17848670

[16] Davis TC Williams MV Marin E Parker RM Glass J. Health literacy and cancer communication. CA Cancer J Clin. (2002) 52:134–49. doi: 10.3322/canjclin.52.3.134 12018928

[17] Scanlon B Brough M Wyld D Durham J. Equity across the cancer care continuum for culturally and linguistically diverse migrants living in Australia: a scoping review. Glob Health. (2021) 17:87. doi: 10.1186/s12992-021-00737-w, PMID: 34321015 PMC8318324

[18] Lillard JW Moses KA Mahal BA George DJ. Racial disparities in Black men with prostate cancer: a literature review. Cancer. (2022) 128:3787–95. doi: 10.1002/cncr.34433, PMID: 36066378 PMC9826514

[19] Aboagye JK Kaiser HE Hayanga AJ. Rural-urban differences in access to specialist providers of colorectal cancer care in the United States: a physician workforce issue. JAMA Surg. (2014) 149:537–43. doi: 10.1001/jamasurg.2013.5062 24740165

[20] Bhatia S Landier W Paskett ED Peters KB Merrill JK Phillips J. Rural–urban disparities in cancer outcomes: opportunities for future research. J Natl Cancer Inst Monogr. (2022) 114:940–52. doi: 10.1093/jnci/djac030, PMID: 35148389 PMC9275775

[21] Cross-Call J. Center on budget and policy priorities: medicaid expansion has helped narrow racial disparities in health coverage and access to care. (2020). Available at: https://www.cbpp.org/research/health/medicaid-expansion-has-helped-narrow-racial-disparities-in-health-coverage-and.

[22] Pierce L. ASCO's commitment to addressing equity, diversity, and inclusion of black cancer patients and survivors. JCO Oncol Pract. (2021) 17:255–7. doi: 10.1200/OP.21.00257, PMID: 33974832

[23] Doak CC Doak LG Friedell GH Meade CD. Improving comprehension for cancer patients with low literacy skills: strategies for clinicians. CA Cancer J Clin. (1998) 48:151–62. doi: 10.3322/canjclin.48.3.151, PMID: 9594918

[24] Patel MI Kapphahn K Dewland M Aguilar V Sanchez B Sisay E. Effect of a community health worker intervention on acute care use, advance care planning, and patient-reported outcomes among adults with advanced stages of Cancer: a randomized clinical trial. JAMA Oncol. (2022) 8:1139–48. doi: 10.1001/jamaoncol.2022.1997, PMID: 35771552 PMC9247857

[25] Kennedy AE Vanderpool RC Croyle RT Srinivasan S. An overview of the National Cancer Institute's initiatives to accelerate rural cancer control research. Cancer Epidemiol Biomarkers Prev. (2018) 27:1240–4. doi: 10.1158/1055-9965.EPI-18-0934, PMID: 30385495 PMC13577081

[26] Surbone A. Cultural competence in oncology: where do we stand? Ann Oncol. (2010) 21:3–5. doi: 10.1093/annonc/mdp546 19940011

[27] Alcaraz KI Wiedt TL Daniels EC Yabroff KR Guerra CE Wender RC eds. Understanding and addressing social determinants to advance cancer health equity in the United States: a blueprint for practice, research, and policy. CA A Cancer J Clin. (2020) 70:31–46. doi: 10.3322/caac.21586 31661164

[28] Patel MI Lopez AM Blackstock W Reeder-Hayes K Moushey EA Phillips J. Cancer disparities and health equity: a policy statement from the American Society of Clinical Oncology. J Clin Oncol. (2020) 38:3439–48. doi: 10.1200/JCO.20.00642, PMID: 32783672 PMC7527158

